# Evolving Antithrombotic Treatment Patterns for Patients With Nonvalvular Atrial Fibrillation and Acute Coronary Syndrome or Underwent Percutaneous Coronary Intervention in China: A Cross-Sectional Study

**DOI:** 10.3389/fcvm.2022.846803

**Published:** 2022-03-18

**Authors:** Ni Suo, Yan-min Yang, Juan Wang, Han Zhang, Xing-hui Shao, Shuang Wu, Jun Zhu

**Affiliations:** The Emergency and Intensive Care Center, State Key Laboratory of Cardiovascular Disease, Fuwai Hospital, National Center for Cardiovascular Diseases, Chinese Academy of Medical Sciences and Peking Union Medical College, Beijing, China

**Keywords:** atrial fibrillation, acute coronary syndromes, PCI–percutaneous coronary intervention, antithrombotic therapy, anticoagulation, antiplatelet

## Abstract

**Objective:**

Antithrombotic therapy in patients with nonvalvular atrial fibrillation (NVAF) concomitant with the acute coronary syndrome (ACS) or underwent percutaneous coronary intervention (PCI) is challenging and has evolved in recent years. However, real-world data on this issue about antithrombotic regimens at discharge and its evolving trend were relatively scarce, especially in China.

**Methods:**

A total of 2,182 patients with NVAF and ACS/PCI were enrolled from 2017 to 2019. A total of 1,979 patients were finally analyzed and divided in three sequential cohorts: cohort 1 (2017), *n* = 674; cohort 2 (2018), *n* = 793; and cohort 3 (2019), *n* = 512. Baseline characteristics and antithrombotic therapy at discharge were analyzed by cohort.

**Results:**

In our cross-sectional study, the majority of patients (59.6%) received dual antiplatelet therapy (DAPT). Over the 3 years, DAPT prescription reduced from nearly 70% to <50% (*P* trend < 0.001), while triple therapy (TT)/double therapy (DT) increased from 27.2 to 50.0% (*P* trend < 0.001). This trend was also seen in different subgroups stratified by CHA2DS2-VASc score, HAS-BLED score, coronary artery disease type, or management type, and was validated after multivariate adjustment. Persistent atrial fibrillation and history of congestive heart failure, hypertension, diabetes mellitus, and stroke/transient ischemic attack/systemic embolism were the independent predictors of TT/DT use, while ACS, PCI, or advanced chronic kidney disease was related with more DAPT prescription.

**Conclusion:**

There is a shift of antithrombotic regime at discharge for patients with NVAF with recent ACS/PCI with reducing DAPT prescription and increasing TT/DT prescription. While the appropriate antithrombotic regimen for patients with NVAF having ACS/PCI is still underused in China.

## Introduction

Atrial fibrillation (AF) is the most common cardiac arrhythmia with increasing prevalence (1) and is associated with a five-fold increase in stroke risk (2). Up to 40% of patients with AF are concomitant with coronary artery disease (CAD) (3). Besides, AF increases the risk of myocardial infarction in patients with and without CAD (4, 5). About 5–15% of patients with AF are known to require percutaneous coronary intervention (PCI) during their entire life (3). Among patients with the acute coronary syndrome (ACS) or undergoing PCI, 2–23% are concomitant with AF (6).

Patients with nonvalvular atrial fibrillation (NVAF) having moderate-to-high stroke risk require chronic oral anticoagulation (OAC) for thromboembolism prevention (7, 8), whereas patients with ACS or undergoing PCI require dual antiplatelet therapy (DAPT) with aspirin and a P2Y12 inhibitor for coronary ischemic events prevention (9). Therefore, combined therapy is needed in patients with NVAF having ACS/PCI. But combined antithrombotic treatment is related to an increased risk of bleeding at the same time (10). The concomitant presence of these conditions represents a challenge in clinical practice.

Antithrombotic therapy of patients concomitant NVAF and ACS/PCI has evolved in recent years with new evidence from pivotal clinical trials in this field (11–14). Evidence-based guidelines and consensus (7, 15–18) recommended a short course of triple therapy (TT) with OAC and DAPT in combination for patients with NVAF after recent ACS/PCI, and following double therapy (DT) with an OAC and single antiplatelet therapy (SAPT) (15, 16). However, observational studies find that patients with AF and ACS/PCI were less likely to receive appropriate antithrombotic therapy (19) and more likely to experience adverse outcomes (20). This study aims to investigate the evolving trends in antithrombotic regimens in Chinese patients with NVAF and ACS/PCI.

## Methods

### Study Design and Participants

**Figure 1:**
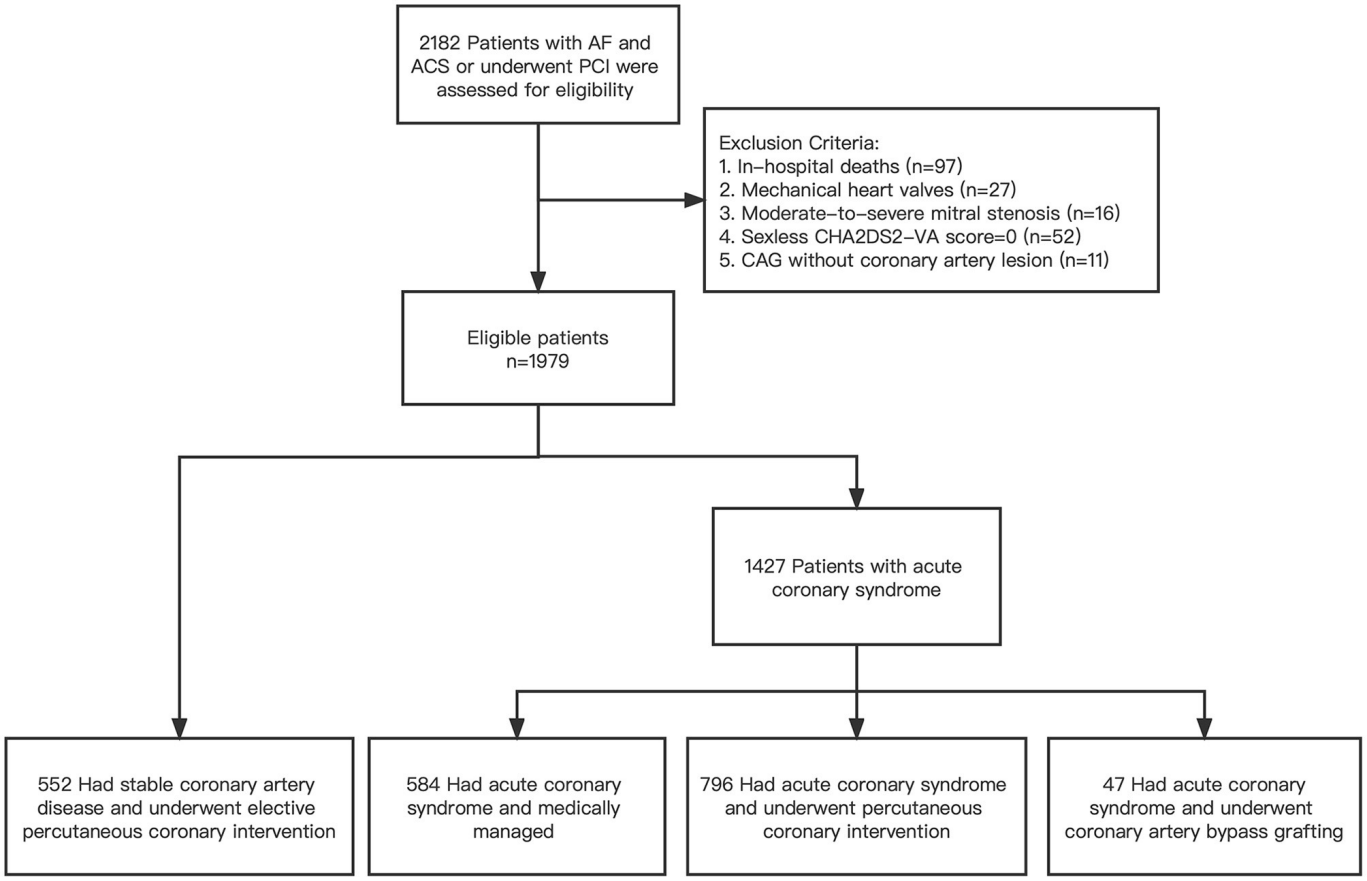
Flowchart of study.

This study is a single-center cross-sectional study of adults with AF and concomitant with ACS or who underwent PCI from 2017 to 2019 in Fuwai Hospital, Beijing, China. Men and women aged over 18 years with AF and ACS or who underwent PCI were enrolled to assess eligibility (Figure 1). At least one of the following risk factors for stroke was required: history of symptomatic heart failure or left ventricular ejection fraction of no more than 40%; hypertension; an age of at least 65 years; diabetes mellitus; previous stroke, transient ischemic attack, or systemic embolism; prior or newly diagnosed acute myocardial infarction; peripheral artery disease with artery stenosis or occlusion. Patients who died in the hospital, patients with valvular AF (i.e., mechanical heart valves, moderate to severe mitral stenosis), and patients who have no atherosclerosis lesion after coronary angiography were excluded. Patients were enrolled consecutively and divided into three sequential cohorts by year: cohort 1 (2017), cohort 2 (2018), and cohort 3 (2019). This article reports cross-sectional data at baseline including antithrombotic therapy pattern at discharge.

### Definitions

The diagnosis of AF was confirmed by reviewing clinical records and electrocardiographic evidence, namely, ECGs, Holter, and rhythm strips. ACS comprised a series of acute coronary diseases, namely, ST-elevation myocardial infarction (STEMI), non-STEMI (NSTEMI), and unstable angina (UA). The diagnosis of ACS was confirmed by clinical physicians based on clinical evaluation, namely, symptoms of cardiac ischemia, 12-lead electrocardiography abnormalities, cardiac biomarkers (cardiac enzyme and cardiac troponin) change, and echocardiography. PCI was defined as percutaneous transluminal coronary revascularization with balloon angioplasty, drug-eluting balloon angioplasty, bare-metal/drug-eluting stent implantation, or unsuccessful coronary intervention attempt.

The definitions of comorbidities are listed as follows. Congestive heart failure was defined with prior symptomatic heart failure and/or left ventricular ejection fraction <40%. Hypertension was defined as a documented history of hypertension or blood pressure over 140/90 mm Hg. Vascular diseases include CAD with prior myocardial infarction (MI), acute MI (AMI), and peripheral artery disease with artery stenosis or occlusion. Prior stroke was defined as a combination of ischemic stroke and hemorrhage stroke.

The CHA2DS2-VASc scores and HAS-BLED scores were used to evaluate the risk of ischemic stroke and bleeding. CHA2DS2-VASc scores were calculated with 1 point for congestive heart failure, hypertension, diabetes, vascular disease, age 65–74 years, and sex category (female), and 2 points for age ≥75 years or stroke. HAS-BLED were calculated with 1 point each for uncontrolled hypertension with systolic blood pressure over 160 mm Hg, abnormal renal function, abnormal hepatic function, previous ischemic or hemorrhagic stroke, bleeding history or predisposition, elderly (age > 65 years), concomitant use of antiplatelet or NSAID and excessive alcohol intake per week, and labile international normalized ratio (INR) is applied for patients receiving warfarin with over 3 times INR measurements. The high risk of ischemic thromboembolism was defined as CHA2DS2-VASc score ≥2, and the high risk of bleeding was defined as HAS-BLED score ≥3.

### Antithrombotic Regimens

The antithrombotic regimens were evaluated by the prescription of antithrombotic drugs at discharge including antiplatelet and oral anticoagulants (OACs). The antiplatelet drugs include aspirin, clopidogrel, and ticagrelor, and OACs comprise both vitamin K antagonist (VKA, e.g., warfarin) and nonvitamin K oral anticoagulants (NOACs, e.g., rivaroxaban or dabigatran).

We classified the antithrombotic treatment according to the combination of prescribed drugs into the following six regimens: no treatment, single antiplatelet therapy (SAPT), dual antiplatelet therapy (DAPT), OAC monotherapy, double therapy (DT, with an OAC and SAPT in combination), and triple therapy (TT, with OAC and DAPT in combination). And TT and DT together are called combined therapy. Besides, TT and DT were further classified into NOAC-based TT (NOAC + DAPT), VKA-based TT (VKA + DAPT), NOAC-based DT (NOAC + SAPT), and VKA-based DT (VKA + SAPT).

### Data Collection

Data about demographic information, baseline comorbidities, and medication usage were collected by trained research personnel *via* interviewing the participants, reviewing medical records (namely, hospital diagnoses and prescription information), and contacting their treating physicians. Individual CHA2DS2-VASc score and HAS-BLED score were calculated by using the information on comorbidities at baseline retrospectively. And source data verification was conducted by the computer-based patient record management system. The study design and protocol have been approved by the Ethics Committee of Fuwai Hospital (Approved No. 2017-923) and conformed to the Declaration of Helsinki. All the patients have signed consent to participate in this study.

### Statistical Analysis

Descriptive statistics were used to summarize and analyze the baseline demographic characteristics, medical history, clinical risk score assessment, and in-hospital invasive treatment by cohort. Besides, the antithrombotic therapy and drug usage were summarized and analyzed by cohort. Then, the temporal trend in antithrombotic therapy was further analyzed by CHA2DS2-VASc score and cohort, by HAS-BLED score and cohort, by CAD type and cohort, and by treatment type and cohort.

To investigate factors affecting the prescription of TT or DT after recent ACS/PCI in patients with AF, baseline demographic characteristics, medical history, clinical risk score, and in-hospital invasive treatment were compared between patients with TT/DT and those with only DAPT. Then logistic regression analysis was performed to investigate the difference in the TT/DT or DAPT prescription across different CHA2DS2-VASc scores and HAS-BLED scores. In addition, multivariable logistic regression analysis was performed using the forward stepwise (LR) method to evaluate the independent predictors of favoring TT/DT over DAPT, with variables comprising year cohort, AF type, CAD type, gender, age, congestive heart failure (CHF), hypertension, diabetes, CAD, PAD, stroke/transient ischemic attack (TIA)/systemic embolism (SE), history of bleeding, advanced chronic kidney disease (CKD) [estimated glomerular filtration rate (eGFR) < 30 ml/min/1.73 m^2^], and in-hospital invasive treatment.

The multivariate logistic regression models were applied to verify the independent effect of time trend on TT/DT use by adjusting for potential confounders. Model 1 was the crude model without adjustment. Model 2 was adjusted for AF type, CAD type, and treatment type. Model 3 was further adjusted factors in the CHA2DS2-VASc score, namely, age, sex, CHF, hypertension, diabetes mellitus, stroke/TIA/SE, prior MI, and peripheral arterial disease.

Continuous variables were expressed as mean ± SD or median with interquartile ranges as appropriate and one-way ANOVA or the Mann–Whitney *U*-test was used to compare the difference among groups. Categorical variables were presented as the frequency with percentage and the chi-squared test was used. The significance of the linear time trend over the study period was assessed using linear regression analysis or logistic regression analysis. Statistical significance was set at *p* < 0.05. All the analyses were performed using software packages SPSS (version 26.0).

## Results

### Study Population

Between January 2017 and December 2019, 2,182 patients with AF and ACS or who underwent PCI were enrolled to assess eligibility. A total of 1,979 patients were included in the final analysis, and divided into three consecutive sequential cohorts by year: cohort 1 (2017), *n* = 674; cohort 2 (2018), *n* = 793; cohort 3 (2019), *n* = 512.

Baseline characteristics and in-hospital treatment strategy were listed in Table 1. Among the baseline characteristics, there is a slightly higher prevalence of ACS, prior CAD, alcohol intake, and a slightly lower prevalence of CHF in cohort 2. During the study period, over 90% of patients were at a high stroke risk with a CHA2DS2-VASc score ≥2, and over 1/3 of patients were at a high bleeding risk with HAS-BLED score ≥3. Considering in-hospital invasive treatment, over two-thirds of patients had undergone PCI and the prevalence of PCI increased over time (*P* trend = 0.014), while the rest variables were similar among the groups.

**Table 1:** Baseline characteristics of the study population.

	**Total**	**Cohort 1**	**Cohort 2**	**Cohort 3**	
**Variable**	**2017–2019**	**2017**	**2018**	**2019**	***p*** **-value**
	***n*** **= 1,979**	***n*** **= 674**	***n*** **= 793**	***n*** **= 512**	
**Demographic characteristics**					
Sex (female sex)	552 (27.9%)	203 (30.1%)	211 (26.6%)	138 (27.0%)	0.281
BMI	25.7 ± 3.4^a^	25.6 ± 3.5^b^	25.7 ± 3.5^c^	25.7 ± 3.2^d^	0.813
Age at diagnosis	67.7 ± 9.6	68.3 ± 9.9	67.4 ± 9.5	67.6 ± 9.5	0.224
**AF type**					
New-onset	166 (8.6%)	57 (8.7%)	69 (8.9%)	40 (8.0%)	0.838
Pre-existing	1,759 (91.4%)	596 (91.3%)	703 (91.1%)	460 (92.0%)	0.838
PAF	1,312 (68.2%)	438 (67.1%)	521 (67.5%)	353 (70.6%)	0.389
PeAF	613 (31.8%)	215 (32.9%)	251 (32.5%)	147 (29.4%)	0.389
**CAD type**					
SCAD	552 (27.9%)	211 (31.3%)	186 (23.5%)	155 (30.3%)	0.001
ACS	1,427 (72.1%)	463 (68.7%)	607 (76.5%)	357 (69.7%)	0.001
**Medical history**					
CHF	414 (20.9%)	139 (20.6%)	131 (16.5%)	144 (28.1%)	<0.001
Hypertension	1,541 (77.9%)	511 (75.8%)	633 (79.8%)	397 (77.5%)	0.179
DM	837 (42.3%)	279 (41.4%)	332 (41.9%)	226 (44.1%)	0.607
STROKE/TIA/SE	513 (25.9%)	177 (26.3%)	212 (26.7%)	124 (24.2%)	0.581
Stroke/TIA	498 (25.2%)	169 (25.1%)	206 (26.0%)	123 (24.0%)	0.728
SE	31 (1.6%)	15 (2.2%)	12 (1.5%)	4 (0.8%)	0.138
PAD	316 (16.0%)	101 (15.0%)	129 (16.3%)	86 (16.8%)	0.670
Prior-CAD	1,347 (68.1%)	444 (65.9%)	566 (71.4%)	337 (65.8%)	0.036
Prior-MI	544 (27.5%)	172 (25.5%)	233 (29.4%)	139 (27.1%)	0.251
Prio-PCI	578 (29.2%)	195 (28.9%)	250 (31.5%)	133 (26.0%)	0.097
Prior-CABG	139 (7.0%)	52 (7.7%)	59 (7.4%)	28 (5.5%)	0.273
Prior-Bleeding	139 (7.0%)	47 (7.0%)	58 (7.3%)	34 (6.6%)	0.896
CKD (eGFR <30 ml/min)	41 (2.1%)	15 (2.2%)	10 (1.3%)	16 (3.1%)	0.066
Cancer	63 (3.2%)	26 (3.9%)	25 (3.2%)	12 (2.3%)	0.338
Current smoker	474 (24.0%)	155 (23.0%)	209 (26.4%)	110 (21.5%)	0.102
Current drinker	438 (22.1%)	155 (23.0%)	196 (24.7%)	87 (17.0%)	0.004
**Clinical risk score**					
CHA2DS2-VASc score	4 (2–5)	4 (3–5)	3 (2–5)	4 (2–5)	0.284
HAS-BLED score	2 (2–3)	2 (2–3)	2 (2–3)	2 (2–3)	0.598
CHA2DS2-VASc score ≥ 2	1,791 (90.5%)	607 (90.1%)	723 (91.2%)	461 (90.0%)	0.706
HAS-BLED score ≥ 3	723 (36.5%)	246 (36.5%)	284 (35.8%)	193 (37.7%)	0.788
**Invasive treatment**					
CAG	1,608 (81.3%)	539 (80.0%)	637 (80.3%)	432 (84.4%)	0.108
PCI	1,348 (68.1%)	444 (65.9%)	531 (67.0%)	373 (72.9%)	0.026
Stent	1,204 (60.8%)	398 (59.1%)	475 (59.9%)	331 (64.6%)	0.115
DEB	96 (4.9%)	24 (3.6%)	46 (5.8%)	26 (5.1%)	0.133
CABG	47 (2.4%)	20 (3.0%)	22 (2.8%)	5 (1.0%)	0.053
None	584 (29.5%)	210 (31.2%)	240 (30.3%)	134 (26.2%)	0.147

a *128 patients missing*.

b *48 patients missing*.

c *40 patients missing*.

d *40 patients missing*.

*Data are presented as number (%) or mean ± SD*.

*AF, atrial fibrillation; PAF, paroxysmal atrial fibrillation; PeAF, persistent atrial fibrillation; SCAD, stable coronary artery disease; ACS, acute coronary syndrome; BMI, body mass index; CHF, congestive heart failure; DM, diabetes mellitus; TIA, transient ischemic attack; SE, systemic embolism; PAD, peripheral arterial disease; CAD, stable coronary artery disease; MI, myocardial infarction; PCI, percutaneous coronary intervention; DEB, drug eluting balloon; CABG, coronary artery bypass graft, CKD, chronic kidney disease; COPD, chronic obstructive pulmonary disease; CAG, coronary angiography*.

### Trend in Antithrombotic Therapy and Drug Usage

Table 2 shows the prescribing pattern at discharge for patients concomitant with NVAF and ACS/PCI in all three cohorts. Although the majority of patients were prescribed DAPT without OAC at discharge, the proportion of DAPT reduced from nearly 70 to <50% over the 3 years (*P* trend < 0.001). The combined therapy (OAC with single or dual antiplatelet) was increased from cohort 1 (27.2%) to cohort 3 (50.0%) (*P* trend < 0.001). Both the proportion of TT (from 14.4 to 30.5%, *P* trend < 0.001) and DT (12.8%−19.5%, *P* trend < 0.001) increased over time. The rise was due to the prevalence use of NOAC, as the prescription of both NOAC + DAPT (from 6.1 to 25.0%, *P* trend < 0.001) and NOAC + SAPT (from 6.5 to 17.6%, *P* trend < 0.001) increased over the study period. At the same time, there was a decline in the use of VKA, regardless of VAK + DAPT (from 8.3 to 5.5%, *P* trend = 0.034) or VKA + SAPT (from 6.2 to 2.0%, *P* trend < 0.001). While the proportion of patients not receiving antithrombotic therapy remained unchanged (*p* = 0.274, *P* trend = 0.161). Considering the drug-specific prescription, there is a decreasing trend in aspirin (from 87.2 to 79.5%, *P* trend < 0.001) and VKA (from 14.7 to 7.6%, *P* trend < 0.001) use. And there is an increasing trend in NOACs (from 13.5 to 42.8%, *P* trend < 0.001) use, especially for rivaroxaban (from 8.5 to 37.3%, *P* trend < 0.001) use. While the prescription rate of clopidogrel, ticagrelor, and dabigatran stayed almost the same.

**Table 2:** Trends of antithrombotic therapy and drug usage of patients with AF concomitant with ACS or underwent PCI.

	**Total**	**Cohort 1**	**Cohort 2**	**Cohort 3**		
**Variable**	**2017–2019**	**2017**	**2018**	**2019**	***p*** **-value**	***P*** -value for trend^*^
	***n*** **= 1,979**	***n*** **= 674**	***n*** **= 793**	***n*** **= 512**		
**Anti-thrombotic therapy**						
OAC + DAPT	423 (21.4%)	97 (14.4%)	170 (21.4%)	156 (30.5%)	<0.001	<0.001
NOAC + DAPT	297 (15.0%)	41 (6.1%)	128 (16.1%)	128 (25.0%)	<0.001	<0.001
VKA + DAPT	126 (6.4%)	56 (8.3%)	42 (5.3%)	28 (5.5%)	0.039	0.034
OAC + SAPT	305 (15.4%)	86 (12.8%)	119 (15.0%)	100 (19.5%)	0.006	0.002
NOAC + SAPT	229 (11.6%)	44 (6.5%)	95 (12.0%)	90 (17.6%)	<0.001	<0.001
VKA + SAPT	76 (3.8%)	42 (6.2%)	24 (3.0%)	10 (2.0%)	<0.001	<0.001
Combined therapy (OAC + DAPT/SAPT)	728 (36.8%)	183 (27.2%)	289 (36.4%)	256 (50.0%)	<0.001	<0.001
OAC	16 (0.8%)	7 (1.0%)	7 (0.9%)	2 (0.4%)	0.446	0.229
DAPT	1,184 (59.8%)	460 (68.2%)	477 (60.2%)	247 (48.2%)	<0.001	<0.001
SAPT	46 (2.3%)	24 (3.6%)	17 (2.1%)	5 (1.0%)	0.013	0.003
None	5 (0.3%)	0 (0.0%)	3 (0.4%)	2 (0.4%)	0.274	0.161
**Drug usage**						
ASA	1,677 (84.7%)	588 (87.2%)	682 (86.0%)	407 (79.5%)	0.001	<0.001
Clopidogrel	1,694 (85.6%)	576 (85.5%)	664 (83.7%)	454 (88.7%)	0.046	0.166
Ticagrelor	194 (9.8%)	60 (8.9%)	84 (10.6%)	50 (9.8%)	0.555	0.564
OAC use	744 (37.6%)	190 (28.2%)	296 (37.3%)	258 (50.4%)	<0.001	<0.001
VKA	206 (10.4%)	99 (14.7%)	68 (8.6%)	39 (7.6%)	<0.001	<0.001
Dabigatran	102 (5.2%)	34 (5.0%)	40 (5.0%)	28 (5.5%)	0.932	0.756
Rivaroxaban	436 (22.0%)	57 (8.5%)	188 (23.7%)	191 (37.3%)	<0.001	<0.001

*OAC, oral anticoagulants; DAPT, dual antiplatelet therapy; SAPT, single antiplatelet therapy; NOAC, nonvitamin K antagonist oral anticoagulants; VKA, vitamin K antagonist*.

* *p value for trend was calculated using linear regression analysis*.

### Subgroup Analysis of Trend in Antithrombotic Therapy

**Figure 2:**
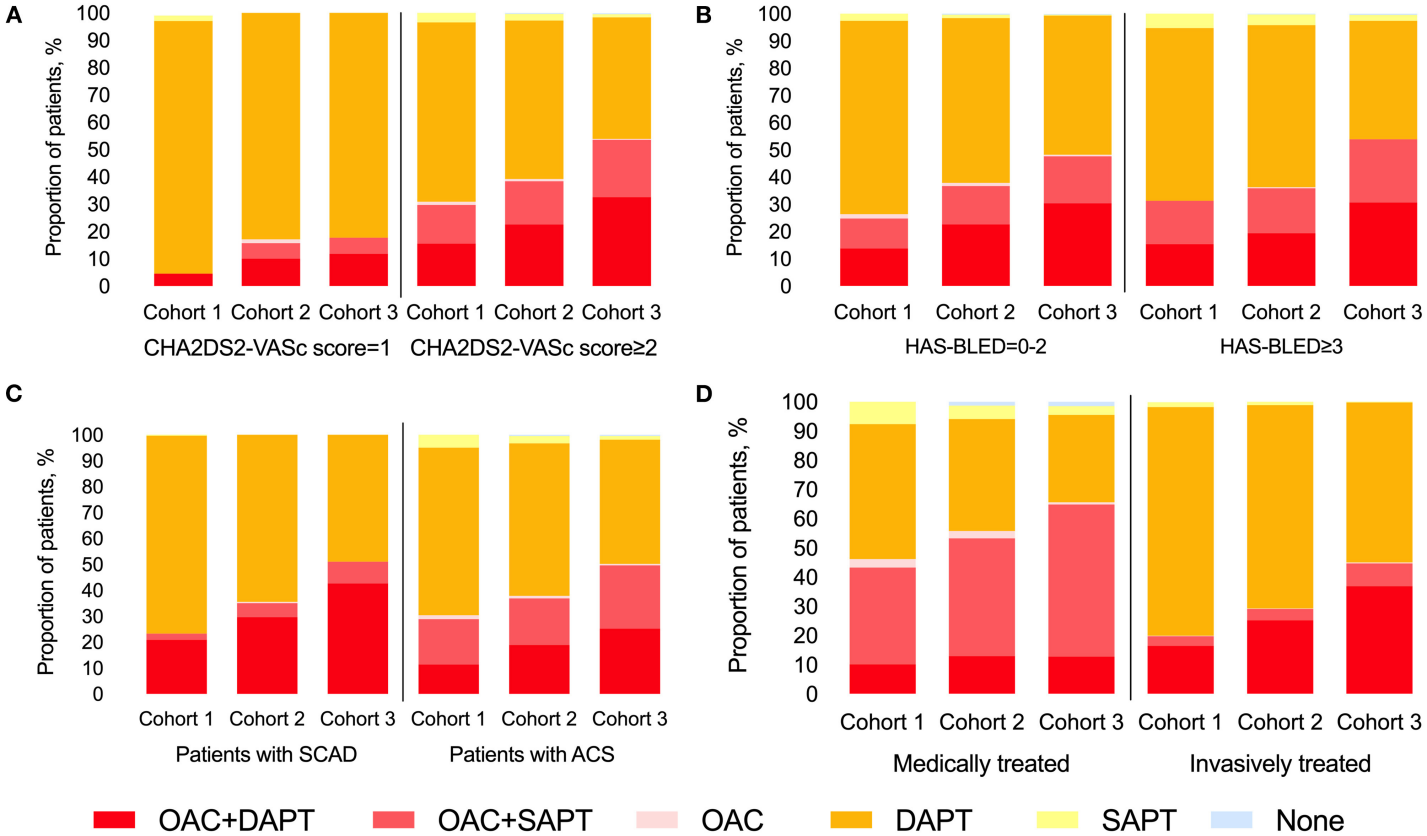
Trend of antithrombotic treatment at discharge stratified by CHA2DS2-VASc score and cohort **(A)**, by HAS-BLED score and cohort **(B)**, by CAD type and cohort **(C)**, and by treatment type and cohort **(D)**. CAD, coronary artery disease; SCAD, stable coronary artery disease; ACS, acute coronary syndrome; OAC, oral anti-coagulants; DAPT, dual antiplatelet therapy; SAPT, single antiplatelet therapy.

**Figure 3:**
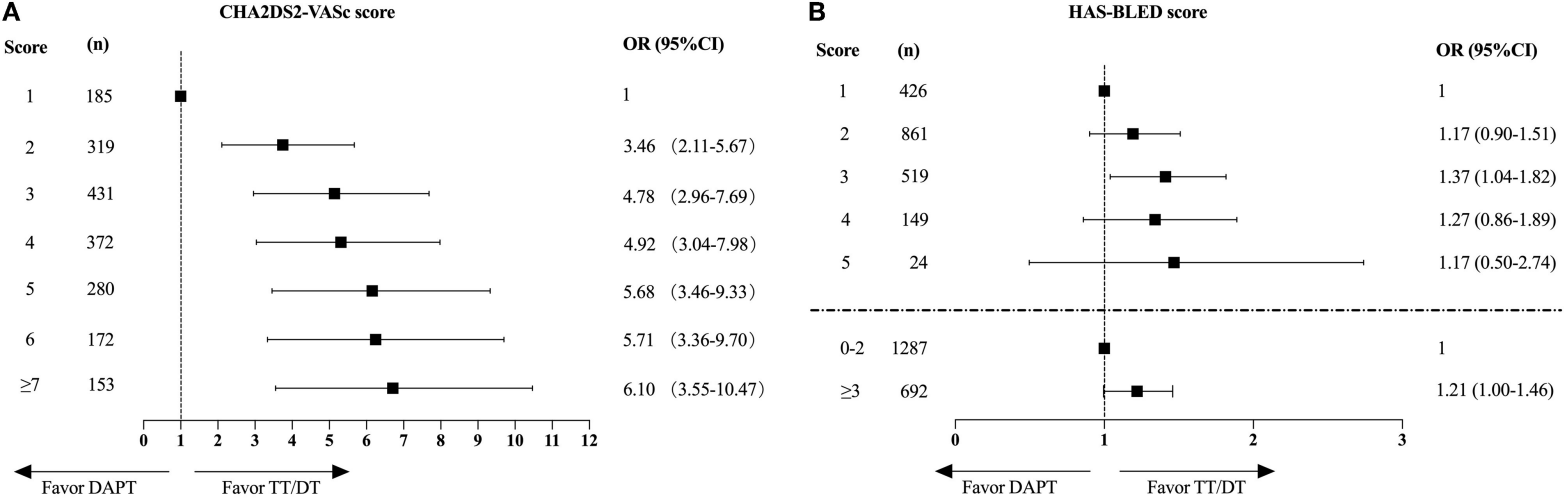
The impact of CHA2DS2-VASc scores **(A)** or HAS-BLED scores **(B)** on the choice of antithrombotic therapy. CHA2DS2-VASc score of 1 and HAS-BLED score of 1 served as the reference respectively. OR, odds ratio; CI, confidence interval; DAPT, dual antiplatelet therapy; TT, triple therapy; DT, double therapy.

The increasing trend of TT/DT prescription was observed regardless of CHA2DS2-VASc score level (Figure 2A), HAS-BLED score level (Figure 2B), CAD type (Figure 2C), or management type (Figure 2D). Patients with CHA2DS2-VASc score ≥2 and patients treated medically had higher TT/DT prescription rates regardless of cohort year when compared with those with CHA2DS2-VASc score = 1 or those treated invasively, while the difference in antithrombotic prescription between patients with different inclusion events (SCAD or ACS) was not obvious. And an incremental relationship was observed between CHA2DS2-VASc score and TT/DT prescription (*P* for trend < 0.001, Figure 3A). While, the proportion of patients on TT/DT stayed the same across different HAS-BLED scores (*P* for trend = 0.058, Figure 3B). When compared with the low bleeding risk subgroup (HAS-BLED score = 0–2), patients with high bleeding risk (HAS-BLED score ≥3) processed a borderline increased likelihood of TT/DT prescription [odds ratio (OR) 1.21, 95%CI 1.00–1.46, Figure 3B].

### Predictors of Favoring TT/DT Over DAPT

Table 3 compares the baseline characteristics and invasive treatment between patients with TT/DT and those with only DAPT. Patients on TT/DT were more likely to be with persistent AF (*p* < 0.001), CHF (*p* < 0.001), hypertension (*p* < 0.001), DM (*p* < 0.001), prior history of stroke/TIA/SE (*p* < 0.001), and possessed higher CHA2DS2-VASc score (*p* < 0.001). Besides, patients with TT/DT were more likely treated conservatively without invasive treatment (*p* < 0.001) and with less stent implantation (*p* < 0.001).

**Table 3:** Baseline characteristics of patients by DATP or TT/DT treatment type.

**Variable**	**DAPT**	**TT/DT**	***p*** **-value**
	***n*** **= 1,184**	***n*** **= 728**	
**Demographic characteristics**			
Sex (female sex)	315 (26.6%)	214 (29.4%)	0.185
BMI	25.6 ± 3.4^a^	25.8 ± 3.4^b^	0.447
Age at diagnosis	67.5 ± 9.9	67.7 ± 8.9	0.565
**AF type**			
New-onset	99 (8.6%)	59 (8.3%)	0.838
Pre-existing	1,053 (91.4%)	650 (91.7%)	0.838
PAF	900 (78.1%)	367 (51.8%)	<0.001
PeAF	252 (21.9%)	342 (48.2%)	<0.001
**CAD type**			
SCAD	357 (30.2%)	193 (26.5%)	0.088
ACS	827 (69.8%)	535 (73.5%)	0.088
**Medical history**			
CHF	162 (13.7%)	231 (31.7%)	<0.001
Hypertension	898 (75.8%)	597 (82.0%)	<0.001
DM	461 (38.9%)	349 (47.9%)	<0.001
STROKE/TIA/SE	263 (22.2%)	227 (31.2%)	<0.001
Stroke/TIA	259 (21.9%)	218 (29.9%)	<0.001
SE	8 (0.7%)	16 (2.2%)	0.004
PAD	174 (14.7%)	130 (17.9%)	0.066
Prior-CAD	821 (69.3%)	486 (66.8%)	0.238
Prior-MI	301 (25.4%)	224 (30.8%)	0.011
Prior-PCI	367 (31.0%)	198 (27.2%)	0.077
Prior-CABG	72 (6.1%)	63 (8.7%)	0.033
Prior-Bleeding	66 (5.6%)	57 (7.8%)	0.051
CKD (eGFR <30 ml/min)	22 (1.9%)	14 (1.9%)	0.919
Cancer	43 (3.6%)	16 (2.2%)	0.078
Current smoker	305 (25.8%)	157 (21.6%)	0.037
Current drinker	279 (23.6%)	148 (20.3%)	0.099
**Clinical risk score**			
CHA2DS2-VASc score	3 (2–5)	4 (3–5)	<0.001
HAS-BLED score	2 (2–3)	2 (2–3)	0.058
**Invasive treatment**			
CAG	1,034 (87.3%)	544 (74.7%)	<0.001
PCI	931 (78.6%)	407 (55.9%)	<0.001
Stent	848 (71.6%)	352 (48.4%)	<0.001
DEB	63 (5.3%)	30 (4.1%)	0.236
PTCA	18 (1.5%)	18 (2.5%)	0.137
CABG	24 (2.0%)	15 (2.1%)	0.96
None	229 (19.3%)	306 (42.0%)	<0.001

a *96 patients missing*.

b *23 patients missing*.

*Data are presented as number (%) or mean ± SD*.

*AF, atrial fibrillation; PAF, paroxysmal atrial fibrillation; PeAF, persistent atrial fibrillation; SCAD, stable coronary artery disease; ACS, acute coronary syndrome; BMI, body mass index; CHF, congestive heart failure; DM, diabetes mellitus; TIA, transient ischemic attack; SE, systemic embolism; PAD, peripheral arterial disease; CAD, stable coronary artery disease; MI, myocardial infarction; PCI, percutaneous coronary intervention; DEB, drug eluting balloon; PTCA, percutaneous transluminal coronary angiography; CABG, coronary artery bypass graft; CKD, chronic kidney disease; COPD, chronic obstructive pulmonary disease; CAG, coronary angiography*.

**Figure 4:**
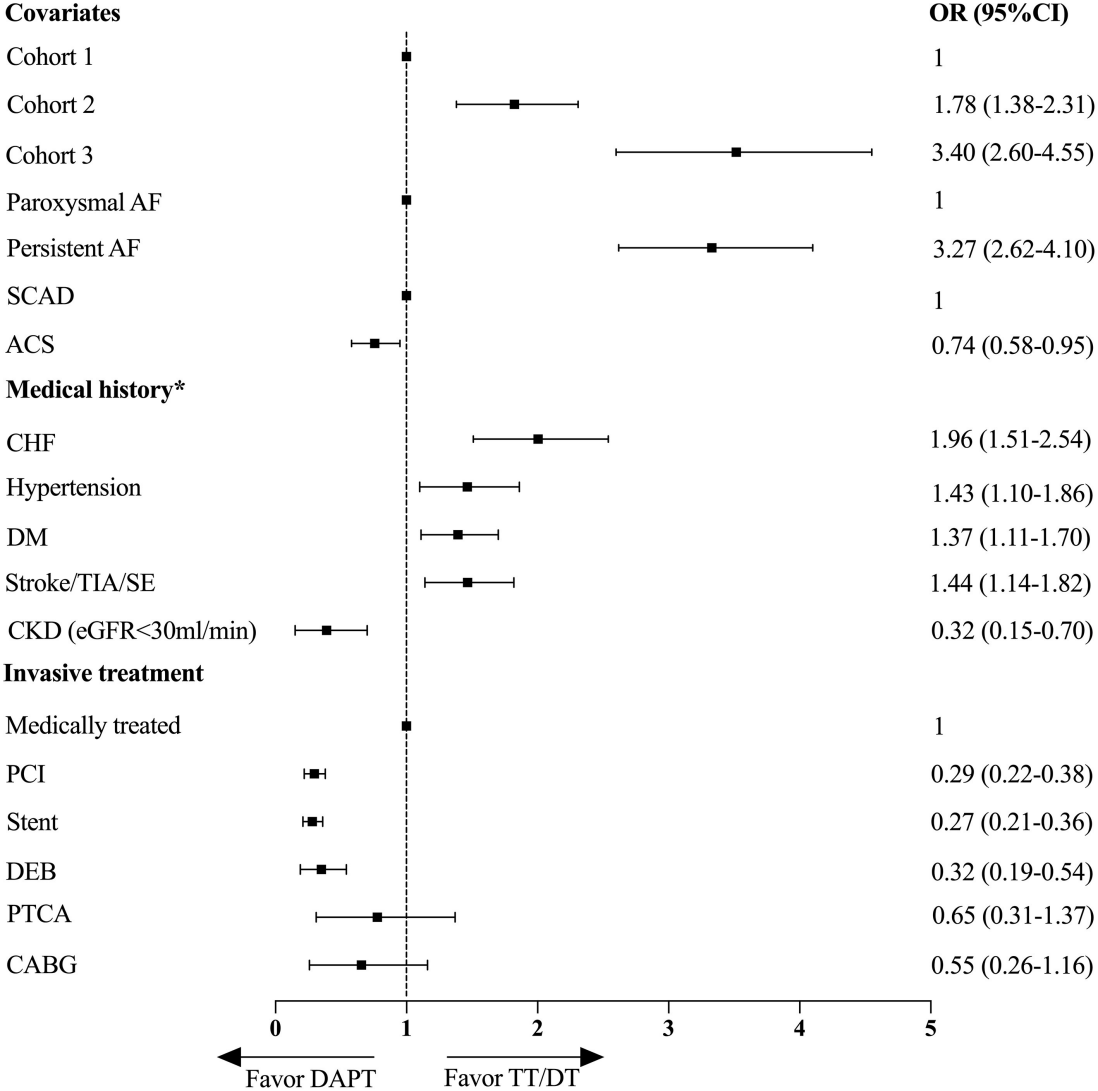
Multivariate logistic regression analysis for factors associated with the prescriptions of TT/DT or DAPT. *Reference group is patient with no medical history or no invasive treatment. OR, odds ratio; CI, confidence interval; DAPT, dual antiplatelet therapy; TT, triple therapy; DT, double therapy; AF, atrial fibrillation; SCAD, stable coronary artery disease; ACS, acute coronary syndrome; CHF, congestive heart failure; DM, diabetes mellitus; TIA, transient ischemic attack; SE, systemic embolism; CKD, chronic kidney disease; PCI, percutaneous coronary intervention; DEB, drug eluting balloon; PTCA, percutaneous transluminal coronary angiography; CABG, coronary artery bypass graft.

Then, the multivariate logistic regression analysis was performed to evaluate the independent predictors associated with the prescription of TT/DT or DAPT (Figure 4). The increased cohort year, persistent AF type, and history of CHF, hypertension, diabetes mellitus, and stroke/TIA/SE were associated with a higher prescription of TT/DT. In addition to increased cohort year, persistent AF was the most significant predictor of TT/DT [oddsratio(OR), 3.27; 95%CI, 2.62–4.10]. While ACS, PCI, or CKD with eGFR <30 ml/min/1.73 m^2^ was associated with underuse of the combined therapy, the most significant predictor of DAPT was PCI (OR, 0.29, 95% CI, 0.22–0.38), while age, sex, history of vascular diseases, and previous bleeding events had little effect on the choice of antithrombotic therapy.

### Verification of Temporal Trend in Evolving Antithrombotic Treatment

The multivariate logistic regression model was applied to verify the independent effect of time trend on TT/DT use. The association between yearly trends and TT/DT use is shown in Table 4. In the crude model, using cohort 1 as a reference, patients in cohort 2 (OR 1.56, 95% CI 1.24–1.96, *p* < 0.001) and cohort 3 (OR 2.73, 95% CI 2.14–3.50, *p* < 0.001) has a higher prescription rate of TT/DT and is increasing over time (*P* trend < 0.001). The results remained statistically significant after adjustment for confounders in Model 2–3. In the full adjusted model, the association between year cohort and TT/DT remained statistically significant (OR 1.76, 95% CI: 1.36–2.27, cohort 2 vs. cohort 1; OR 3.43, 95% CI: 2.59–4.54, cohort 3 vs. cohort 1; *P* trend < 0.001).

**Table 4:** Odds ratio (OR) and 95%CI for TT/DT antithrombotic treatment according to year cohort.

	**Cohort 1**	**Cohort 2**	**Cohort 3**	
	**2017**	**2018**	**2019**	***P*** **-value**
	***n*** **= 674**	***n*** **= 793**	***n*** **= 512**	for trend^*^
Model 1:	1.00 (Ref)	1.56 (1.24 to 1.96)	2.73 (2.14 to 3.50)	<0.001
*p*-values		<0.001	<0.001	
Model 2	1.00 (Ref)	1.77 (1.38 to 2.26)	3.57 (2.73 to 4.67)	<0.001
*p*-values		<0.001	<0.001	
Model 3	1.00 (Ref)	1.76 (1.36 to 2.27)	3.43 (2.59 to 4.54)	<0.001
*p*-values		<0.001	<0.001	

*Association between year cohort with TT/DT antithrombotic treatment*.

*Model 1 was crude model, with no adjustment. Model 2 was adjusted for AF type, CAD type and treatment type. Model 3 was further adjusted for factors in CHA2DS2-VASc score, namely, age, sex, CHF, hypertension, diabetes mellitus, stroke/TIA/SE, prior-myocardial infarction, and peripheral arterial disease*.

* *p-value for trend was calculated using logistic regression analysis*.

## Discussion

In this study, we investigated the evolving trend in antithrombotic therapy, and our principal findings were as follows: (1) The majority of patients were administered DAPT without OAC; (2) Both the TT and DT prescriptions showed a gradual increment over time, and the total amount of TT/DT prescriptions surpassed 50% in 2019; (3) An incremental relationship between CHA2DS2-VASc score and combined therapy prescription was identified; (4) The predictors of TT/DT use were persistent AF type and history of CHF, hypertension, diabetes mellitus, and stroke/TIA/SE. ACS or PCI and CKD with eGFR <30 ml/min/1.73 m^2^ were associated with underuse of the combined therapy; and (5) Temporal trend was the independent factor related to the incremental use of TT/DT.

The choice of antithrombotic therapy for patients concomitant of AF and ACS/PCI presents a great challenge in the real-world clinical scenario. The risk of ischemic events and the risk of bleeding need to be balanced carefully. Plenty of efforts have been made to explore this issue since 2013. Based on the result of WOEST (21), ISAR-TRIPLE (22), and four pivotal NOAC-based RCTs (11–14), guidelines and consensus documents updated from the North American (16, 17), European (7, 15), and Chinese (18) recommended combined therapy with short-course TT and following DT in patients with NVAF and ACS/PCI. Besides, NOACs are preferred over VKA, and clopidogrel is the most advocated P2Y12 inhibitor.

However, the data on detailed temporal trends in real-world clinical practice were relatively scarce, especially in China. Our study shows a clear gap between real-world everyday medical practice and guideline recommendations. In our study, DAPT was the most commonly prescribed regimen at discharge (59.8%), rather than the guideline-recommended TT/DT. And there is a small proportion of patients were prescribed with merely OAC, SAPT, or no treatment. These results highlight the undertreated status of patients with NVAF and ACS/PCI in China and are in line with other countries. A Korean study (23) reported their nationwide registries from 2013 to 2018, and OAC in patients with AF after PCI were underprescribed. Besides, this phenomenon was also prevalent in western populations. A nationwide Danish study (24) retrospectively analyzed the antithrombotic treatment of 8,659 patients with AF admitted with a MI (*n* = 6,362) and/or underwent PCI (*n* = 2,297) from 2011 to 2017, finding <1/2 patients were treated with DT/TT (*n* = 3,222). The underprescription of OAC-based TT/DT in our study might be caused by concerns of physicians in China. As TT/DT has been shown to increase the risk of bleeding (25), Asians are more prone to suffer from anticoagulant-related bleeding and intracranial hemorrhage when compared with Caucasians (26, 27). For the sake of safety, physicians were hesitant to add OAC on top of antiplatelet therapy. Bedsides, this grim status is partially related to the insufficient awareness of thromboembolism risk and exaggerated concern of bleeding risk in Chinese patients with AF (28). The need for regular INR monitoring and excessive interaction with other medication or foods were related to the reluctance of patients to receive warfarin. Although NOACs have been proved to possess a favorable safety, efficacy, and convenience, the less well-off economic status and insufficient healthcare expenditure limited the wide application of NOACs in China (29). These indicate that better academic education and health policies are required to improve the management status for patients with AF and ACS/PCI.

Then, we investigate the difference in the TT/DT or DAPT prescription across different CHA2DS2-VASc scores and HAS-BLED scores by utilizing logistic regression analysis. The CHA2DS2-VASc score was significantly related to the prescription of TT/DT, and the association was more pronounced in higher risk score subgroups. While, the bleeding risk evaluated by HAS-BLED score has little effect on the choice of antithrombotic regimens, as the proportion of patients on TT/DT stayed almost the same across different HAS-BLED scores. Besides, patients with higher bleeding risk (HAS-BLED score ≥3) possessed a borderline increased likelihood of TT/DT prescription. This might due to the fact that bleeding risk factors frequently overlap with thromboembolism risk factors. Those patients with high bleeding risk often possess high thromboembolic risk as well. Besides, clinical guidelines suggest that HAS-BLED scores should be utilized to assess the risk of bleeding, rather than define whether a patient should be treated with OAC (16). By using the HAS-BLED score, potentially modifiable bleeding risk factors can be identified and altered by treatment or by changing lifestyle. So, the prescription rate of TT/DT did not decrease in patients with high bleeding risk (HAS-BLED score ≥3) in this study.

Moreover, we explored the independent predictors of TT/DT prescription and the factors associated with the suboptimal use of combined therapy by using multivariable logistic regression analysis. In addition to the temporal trend of TT/DT use, persistent AF type and history of CHF, hypertension, diabetes mellitus, and stroke/TIA/SE were associated with a higher prescription of TT/DT. This is in line with a previous study utilizing Korea nationwide registry data, which also found traditional thromboembolism risk factors, namely, female gender, DM, prior cerebrovascular accident, and CHF are significant determinants of OAC use (30). And patients with persistent AF possessed higher thromboembolism risk when compared with those with paroxysmal AF (31). Our results indicate that more AF burden and traditional thromboembolism risk factors have more impact on clinical antithrombotic strategy making, and patients with these factors were more inclined to receive combined therapy. While ACS or PCI and advanced CKD stage were associated with underuse of the combined therapy. The prevalent use of DAPT for patients with NVAF having recent coronary ischemic events or coronary intervention indicates intensive attention was on coronary ischemic events prevention and insufficient awareness was on thromboembolism prophylaxis. As DAPT is the default treatment to prevent coronary ischemic events after PCI, and TT/DT increased the risk of bleeding compared with DAPT, physicians were hesitant to add OAC on top of antiplatelet therapy. With evolving evidence and guidelines recommendations, more and more physicians gradually realize that DAPT alone was not sufficient for thromboembolism prevention. This situation has been gradually improved during our study period with an increasing trend of TT/DT prescription. Although CKD is a prothrombotic and prohemorrhagic factor among patients with AF. Anticoagulant therapy was associated with a decreased risk of stroke or systemic thromboembolism among patients concomitant with AF and CKD (32). While, this is still a conundrum in clinical practice, and associated with the underuse of combined therapy. Knowledge regarding these factors related to the selection of antithrombotic therapy is important and necessary for further improving the treatment for patients with NVAF having ACS/PCI.

Another important finding in this study was that a pronounced change in the pattern of antithrombotic therapy prescription has been made in China. There is a shift of antithrombotic regime with reducing DAPT prescription and increasing combined therapy (TT/DT) prescription. This trend was also seen in different subgroups stratified by CHA2DS2-VASc score, HAS-BLED score, CAD type (ACS or SCAD), or management type (invasive treated or medically treated). Moreover, this trend stayed validated after adjusting for confounders, namely, AF type, CAD type, and treatment type, factors in the CHA2DS2-VASc score. This phenomenon demonstrated that clinical practice has been influenced by evolving evidence and guidelines recommendations, which is in line with the result from Taiwan (33). This paradigm shift in prescribing practice has been driven in part by the prevalence use of NOACs with a better risk-to-benefit ratio of NOAC-based DT than VKA-based TT (34). In addition, this trend might also be driven by the realization that DAPT alone was not sufficient for thromboembolism prevention (25).

### Strengths

There are several strengths in this study. First, this study was conducted in the largest medical center for cardiovascular diseases in China, representing the most advanced level of clinical practice in our country. To the best of our knowledge, this is the largest cross-sectional study conducted in China until now in this field. Second, a great deal of clinically important information was collected, namely, patient baseline characteristics, previous history, bleeding profile, and specific treatment strategy. In addition, source data verification was conducted by the computer-based patient record management system to ensure validity.

### Limitations

This study also has some limitations. First, since our data were derived from a single medical center, there may be a discrepancy between the drug prescription habit of this single center and the overall population of China. Generalizing the antithrombotic utilization patterns beyond this level of healthcare setting warrants caution. Second, the prescription of TT/DT might be overestimated as some OAC prescription was due to transient purposes like cardioversion or AF ablation. Third, patients with AF may have some specific angiographic characteristics (35). The severity of coronary lesions may influence the choice of antithrombotic regimen. While in this study, procedure-related characteristics in PCI or CABG were not documented and we cannot conduct further relevant analysis. In addition, this study shares the limitations of the cross-sectional study. Duration of therapy, switch of antithrombotic regimens, and clinical outcomes, namely, recurrent thrombotic events, major bleeding events, mortality, and other major adverse clinical events for each regimen were not evaluated. Besides, as NOACs are associated with reduced major adverse outcomes in patients with NVAF (36), future studies can focus on and further investigate the difference in clinical outcome between those treated with NOACs and those treated with VKA in patients with NVAF and ACS/PCI. Lastly, there could still be residual confounding due to unmeasured variables or inadequate control, although substantial efforts have been made in variable collection and adjustments.

## Conclusion

The antithrombotic treatment at discharge for patients with NVAF having recent ACS/PCI has noticeably changed in recent years. There is a shift of antithrombotic regimen with reducing DAPT prescription and increasing combined therapy (TT/DT) prescription. The predictors of TT/DT use ware persistent AF type and history of CHF, hypertension, diabetes mellitus, and stroke/TIA/SE, while ACS or PCI and CKD with eGFR <30 ml/min/1.73 m^2^ were associated with underuse of the combined therapy. Further studies are warranted to elucidate the clinical outcome with different antithrombotic treatment regimes in Chinese real-world clinical practice.

## Data Availability Statement

The raw data supporting the conclusions of this article will be made available by the authors, without undue reservation.

## Ethics Statement

The studies involving human participants were reviewed and approved by the Ethics Committee of Fuwai Hospital (Approved No. 2017-923) and conformed to the Declaration of Helsinki. The patients/participants provided their written informed consent to participate in this study.

## Author Contributions

NS collected the data, performed the statistical analysis, drafted, and wrote the manuscript. Y-mY and JZ designed the study and revised the manuscript. JW, HZ, X-hS, and SW collected the data. All authors read and approved the final manuscript.

## Funding

This work was supported by Capital's Funds for Research and Application of Clinical Diagnosis and Treatment Technology (Grant/Award Number: Z191100006619121).

## Conflict of Interest

The authors declare that the research was conducted in the absence of any commercial or financial relationships that could be construed as a potential conflict of interest.

## Publisher's Note

All claims expressed in this article are solely those of the authors and do not necessarily represent those of their affiliated organizations, or those of the publisher, the editors and the reviewers. Any product that may be evaluated in this article, or claim that may be made by its manufacturer, is not guaranteed or endorsed by the publisher.

## References

[1] Virani SS Alonso A Benjamin EJ Bittencourt MS Callaway CW Carson AP. Heart disease and stroke statistics-2020 update: a report from the American Heart Association. Circulation. (2020) 141:e139–596. 10.1161/CIR.0000000000000746 31992061

[2] Wolf PA Abbott RD Kannel WB. Atrial fibrillation as an independent risk factor for stroke: the Framingham Study. Stroke. (1991) 22:983–8. 10.1161/01.STR.22.8.983 1866765

[3] Michniewicz E Mlodawska E Lopatowska P Tomaszuk-Kazberuk A Malyszko J. Patients with atrial fibrillation and coronary artery disease – double trouble. Adv Med Sci. (2018) 63:30–5. 10.1016/j.advms.2017.06.005 28818746

[4] Fanaroff AC Li S Marquis-Gravel G Giri J Lopes RD Piccini JP. Atrial fibrillation and coronary artery disease: a long-term perspective on the need for combined antithrombotic therapy. Circ Cardiovasc Interv. (2021) 14:e011232. 10.1161/CIRCINTERVENTIONS.121.011232 34932388

[5] Ruddox V Sandven I Munkhaugen J Skattebu J Edvardsen T Otterstad JE. Atrial fibrillation and the risk for myocardial infarction, all-cause mortality and heart failure: a systematic review and meta-analysis. Eur J Prev Cardiol. (2017) 24:1555–66. 10.1177/2047487317715769 28617620 PMC5598874

[6] Gonzalez-Pacheco H Marquez MF Arias-Mendoza A Alvarez-Sangabriel A Eid-Lidt G Gonzalez-Hermosillo A. Clinical features and in-hospital mortality associated with different types of atrial fibrillation in patients with acute coronary syndrome with and without ST elevation. J Cardiol. (2015) 66:148–54. 10.1016/j.jjcc.2014.11.001 25480145

[7] Hindricks G Potpara T Dagres N Arbelo E Bax JJ Blomstrom-Lundqvist C. 2020 ESC Guidelines for the diagnosis and management of atrial fibrillation developed in collaboration with the European Association for Cardio-Thoracic Surgery (EACTS): the task force for the diagnosis and management of atrial fibrillation of the European Society of Cardiology (ESC) Developed with the special contribution of the European Heart Rhythm Association (EHRA) of the ESC. Eur Heart J. (2021) 42:373–498. 10.1093/eurheartj/ehab648 32860505

[8] Writing Group M January CT Wann LS Calkins H Chen LY Cigarroa JE. 2019 AHA/ACC/HRS focused update of the 2014 AHA/ACC/HRS guideline for the management of patients with atrial fibrillation: a report of the American College of Cardiology/American Heart Association Task Force on Clinical Practice Guidelines and the Heart Rhythm Society. Heart Rhythm. (2019) 16:e66–93. 10.1016/j.hrthm.2019.01.024 30703530

[9] Capodanno D Alfonso F Levine GN Valgimigli M Angiolillo DJ. ACC/AHA versus ESC guidelines on dual antiplatelet therapy: JACC guideline comparison. J Am Coll Cardiol. (2018) 72(23 Pt A):2915–31. 10.1016/j.jacc.2018.09.057 30522654

[10] Buccheri S Angiolillo DJ Capodanno D. Evolving paradigms in antithrombotic therapy for anticoagulated patients undergoing coronary stenting. Ther Adv Cardiovasc Dis. (2019) 13:1753944719891688. 10.1177/1753944719891688 31814532 PMC6902384

[11] Gibson CM Mehran R Bode C Halperin J Verheugt FW Wildgoose P. Prevention of bleeding in patients with atrial fibrillation undergoing PCI. N Engl J Med. (2016) 375:2423–34. 10.1056/NEJMoa1611594 27959713

[12] Cannon CP Bhatt DL Oldgren J Lip GYH Ellis SG Kimura T. Dual antithrombotic therapy with dabigatran after PCI in atrial fibrillation. N Engl J Med. (2017) 377:1513–24. 10.1056/NEJMoa1708454 28844193

[13] Lopes RD Heizer G Aronson R Vora AN Massaro T Mehran R. Antithrombotic therapy after acute coronary syndrome or PCI in atrial fibrillation. N Engl J Med. (2019) 380:1509–24. 10.1056/NEJMoa1817083 30883055

[14] Vranckx P Valgimigli M Eckardt L Tijssen J Lewalter T Gargiulo G. Edoxaban-based versus vitamin K antagonist-based antithrombotic regimen after successful coronary stenting in patients with atrial fibrillation (ENTRUST-AF PCI): a randomised, open-label, phase 3b trial. Lancet. (2019) 394:1335–43. 10.1016/S0140-6736(19)31872-0 31492505

[15] Lip GYH Collet JP Haude M Byrne R Chung EH Fauchier L. 2018 Joint European consensus document on the management of antithrombotic therapy in atrial fibrillation patients presenting with acute coronary syndrome and/or undergoing percutaneous cardiovascular interventions: a joint consensus document of the European Heart Rhythm Association (EHRA), European Society of Cardiology Working Group on Thrombosis, European Association of Percutaneous Cardiovascular Interventions (EAPCI), and European Association of Acute Cardiac Care (ACCA) endorsed by the Heart Rhythm Society (HRS), Asia-Pacific Heart Rhythm Society (APHRS), Latin America Heart Rhythm Society (LAHRS), and Cardiac Arrhythmia Society of Southern Africa (CASSA). Europace. (2019) 21:192–3. 10.1093/europace/euy174 30052888

[16] Angiolillo DJ Bhatt DL Cannon CP Eikelboom JW Gibson CM Goodman SG. Antithrombotic therapy in patients with atrial fibrillation treated with oral anticoagulation undergoing percutaneous coronary intervention: a North American perspective: 2021 update. Circulation. (2021) 143:583–96. 10.1161/CIRCULATIONAHA.120.050438 33555916

[17] January CT Wann LS Calkins H Chen LY Cigarroa JE Cleveland JC Jr.. 2019 AHA/ACC/HRS focused update of the 2014 AHA/ACC/HRS guideline for the management of patients with atrial fibrillation: a report of the American College of Cardiology/American Heart Association Task Force on Clinical Practice Guidelines and the Heart Rhythm Society. J Am Coll Cardiol. (2019) 74:104–32. 10.1016/j.jacc.2019.01.011 30703431

[18] Chinese Chinese Society of Cardiolgy of Chinese Medical A Editorial Editorial Board of Chinese Journal of C. Antithrombotic management of patients with atrial fibrillation and coronary artery disease: expert consensus document of Chinese Society of Cardiology. Zhonghua Xin Xue Guan Bing Za Zhi. (2020) 48:552–64. 10.3760/cma.j.cn112148-20200328-00257 32842267

[19] Guimaraes PO Zakroysky P Goyal A Lopes RD Kaltenbach LA Wang TY. Usefulness of antithrombotic therapy in patients with atrial fibrillation and acute myocardial infarction. Am J Cardiol. (2019) 123:12–8. 10.1016/j.amjcard.2018.09.031 30409413

[20] Erez A Goldenberg I Sabbag A Nof E Zahger D Atar S. Temporal trends and outcomes associated with atrial fibrillation observed during acute coronary syndrome: Real-world data from the Acute Coronary Syndrome Israeli Survey (ACSIS), 2000-2013. Clin Cardiol. (2017) 40:275–80. 10.1002/clc.22654 27918068 PMC6490420

[21] Dewilde WJ Oirbans T Verheugt FW Kelder JC De Smet BJ Herrman JP. Use of clopidogrel with or without aspirin in patients taking oral anticoagulant therapy and undergoing percutaneous coronary intervention: an open-label, randomised, controlled trial. Lancet. (2013) 381:1107–15. 10.1016/S0140-6736(12)62177-1 23415013

[22] Fiedler KA Maeng M Mehilli J Schulz-Schupke S Byrne RA Sibbing D. Duration of triple therapy in patients requiring oral anticoagulation after drug-eluting stent implantation: the ISAR-TRIPLE trial. J Am Coll Cardiol. (2015) 65:1619–29. 10.1016/j.jacc.2015.02.050 25908066

[23] Kwon S Jung JH Choi EK Lee SW Park J Lee SR. Impact of non-vitamin K antagonist oral anticoagulants on the change of antithrombotic regimens in patients with atrial fibrillation undergoing percutaneous coronary intervention. Korean Circ J. (2021) 51:409–22. 10.4070/kcj.2020.0407 33764010 PMC8112178

[24] Sindet-Pedersen C Lamberts M Staerk L Nissen Bonde A Berger JS Pallisgaard JL. Combining oral anticoagulants with platelet inhibitors in patients with atrial fibrillation and coronary disease. J Am Coll Cardiol. (2018) 72:1790–800. 10.1016/j.jacc.2018.07.054 30286922

[25] Lamberts M Gislason GH Olesen JB Kristensen SL Schjerning Olsen AM Mikkelsen A. Oral anticoagulation and antiplatelets in atrial fibrillation patients after myocardial infarction and coronary intervention. J Am Coll Cardiol. (2013) 62:981–9. 10.1016/j.jacc.2013.05.029 23747760

[26] Sabir I Khavandi K Brownrigg J Camm AJ. Oral anticoagulants for Asian patients with atrial fibrillation. Nat Rev Cardiol. (2014) 11:290–303. 10.1038/nrcardio.2014.22 24614113

[27] Shen AY Yao JF Brar SS Jorgensen MB Chen W. Racial/ethnic differences in the risk of intracranial hemorrhage among patients with atrial fibrillation. J Am Coll Cardiol. (2007) 50:309–15. 10.1016/j.jacc.2007.01.098 17659197

[28] Sun Y Wang Y Jiang J Wang L Hu D. Renal dysfunction, CHADS2 score, and adherence to the anticoagulant treatment in nonvalvular atrial fibrillation. Clin Appl Thromb Hemost. (2017) 23:248–54. 10.1177/1076029615611250 26467322

[29] Chang SS Dong JZ Ma CS Du X Wu JH Tang RB. Current status and time trends of oral anticoagulation use among Chinese patients with nonvalvular atrial fibrillation: the Chinese atrial fibrillation registry study. Stroke. (2016) 47:1803–10. 10.1161/STROKEAHA.116.012988 27283198

[30] Lee OH Kim Y Cho DK Kim JS Kim BK Choi D. Temporal trends of antithrombotic therapy in patients with acute myocardial infarction and atrial fibrillation: insight from the KAMIR-NIH registry. Front Cardiovasc Med. (2021) 8:762090. 10.3389/fcvm.2021.762090 34901221 PMC8655723

[31] Ren J Yang Y Zhu J Wu S Wang J Zhang H. Type of atrial fibrillation and outcomes in patients without oral anticoagulants. Clin Cardiol. (2021) 44:168–75. 10.1002/clc.23519 33314221 PMC7852164

[32] Olesen JB Lip GY Kamper AL Hommel K Kober L Lane DA. Stroke and bleeding in atrial fibrillation with chronic kidney disease. N Engl J Med. (2012) 367:625–35. 10.1056/NEJMoa1105594 22894575

[33] Lee CC Chang CH Hung Y Lin CS Yang SP Cheng SM. Changes of antithrombotic prescription in atrial fibrillation patients with acute coronary syndrome or percutaneous coronary intervention and the subsequent impact on long-term outcomes: a longitudinal cohort study. Thromb J. (2021) 19:100. 10.1186/s12959-021-00353-z 34906162 PMC8670061

[34] Zhao S Hong X Cai H Liu M Li B Ma P. Antithrombotic management for atrial fibrillation patients undergoing percutaneous coronary intervention or with acute coronary syndrome: an evidence-based update. Front Cardiovasc Med. (2021) 8:660986. 10.3389/fcvm.2021.660986 34262952 PMC8273244

[35] Pastori D Biccire FG Lip GYH Menichelli D Pignatelli P Barilla F. Relation of atrial fibrillation to angiographic characteristics and coronary artery disease severity in patients undergoing percutaneous coronary intervention. Am J Cardiol. (2021) 141:1–6. 10.1016/j.amjcard.2020.11.006 33220321

[36] Pastori D Menichelli D Del Sole F Pignatelli P Violi F. Long-term risk of major adverse cardiac events in atrial fibrillation patients on direct oral anticoagulants mayo. Clin Proc. (2021) 96:658–65. 10.1016/j.mayocp.2020.06.057 33308867

